# The effect of mild agonist stimulation on the platelet reactivity in patients with type 2 diabetes mellitus

**DOI:** 10.1186/s12902-019-0391-2

**Published:** 2019-06-14

**Authors:** Razie Mahmoodian, Morteza Salimian, Mohsen Hamidpour, Ali Akbar Khadem-Maboudi, Ahmad Gharehbaghian

**Affiliations:** 1 Department of Hematology and Blood Banking, School of Allied Medical Sciences, Shahid Beheshti University of Medical Sciences, Tehran, 1971653313 Iran; 2 Paramedical Faculty, Kashan University of Medical Sciences, Kashan, Iran; 3 Department of Hematology and Blood Banking, School of Allied Medical Sciences, Shahid Beheshti University of Medical Sciences, Tehran, Iran; 4 Department of Bio statistical, School of Paramedical Sciences, Shahid Beheshti University of Medical Sciences, Tehran, Iran; 5 Department of Hematology and Blood Banking, School of Allied Medical Sciences, Shahid Beheshti University of Medical Sciences, Tehran, Iran

**Keywords:** Type 2 diabetes, Desensitization, Adenosine diphosphate (ADP), Platelets

## Abstract

**Background:**

Patients with type 2 diabetes mellitus (T2DM) have accelerated atherosclerosis as a pro thrombotic state that is associated with the platelet activation priming. Platelets, which undergo the continuous mild stimulation, may lose their sensitivity to react to a strong stimulation. The present study aimed to investigate activation responses of platelets to mild and subsequent strong stimulations in patients with T2DM and healthy individuals.

**Methods:**

Blood samples, which were taken from 40 patients with T2DM and 35 healthy individuals, were collected into the citrate containing tubes. The samples were subjected to the soft centrifugation to prepare the platelet rich plasma (PRP). Platelets in PRP samples were treated at a low (1 μM) concentration and then at a high (10 μM) concentration of ADP. Before and after stimulation with different doses of ADP, levels of CD62P expression and formation of platelet micro particles (PMPs) were measured using a flow cytometry method.

**Results:**

The platelets from patients with T2DM had higher levels of CD62P expression before any stimulation (*P* = 0.003) than control samples. Platelets, which underwent the mild stimulation, indicated lower responses to CD62P expression, but higher PMPs formation after stimulation with high dose of ADP. Patients with T2DM had higher platelet micro particles in all states with the ADP stimulation. (*P* = 0.004, SD: ±74.52).

**Conclusions:**

The flow cytometry data indicated that platelets were pre-active and associated with metabolic conditions in patients with type 2 diabetes mellitus. The induction of desensitization state helped platelets to reduce the platelet activation and sensitivity to ADP in a diabetic environment. Furthermore, the production of platelets micro-particles was high in the patients; and desensitized platelets were more susceptible to shedding of micro-particles.

## Background

Diabetes is a group of metabolic diseases that can be diagnosed by Hyperglycemia and is due to impaired insulin secretion, insulin performance, or both [1]. Type 2 diabetes mellitus (T2DM) is the cause of over 80% of diabetes mellitus cases and emerges as a result of genetic and environmental factors. Impaired insulin secretion and reduced sensitivity to insulin in the liver tissue, skeleton muscles and fat tissue are main pathophysiological characteristics of T2DM [2]. Patients with T2DM have higher risk of developed atherosclerosis. [3]. Hyperglycemia and several other factors including hypercoagulability, impaired fibrinolysis system, impaired endothelial cells, hyperactive platelets and production of platelet micro-particles (PMPs) contribute in the creation of a pro-thrombotic state in patients with T2DM. [4, 5]. Several factors are involved in the effective formation of hyperactive platelets in T2DM [6, 7] including hyperglycemia, insulin deficiency, insulin resistance [8], increased systemic inflammation, some metabolic conditions such as obesity and dyslipidemia, some cellular disorders such as failure in regulating calcium metabolism, increasing the oxidative stress, and increasing the P2Y12 signaling and the platelet turnover [9].

Platelet micro-particles (PMPs) are small cellular fragments within the range of 0.1 to 1 μm that are separated from the membrane of activated platelets, stressed platelets, and apoptotic platelets through shedding. They have phospholipid plasma membrane and inherit a variety of functional receptors from platelets [10, 11]. These cellular fragments are considered to be the most abundant blood micro-particles expressing some pre-coagulant molecules such as phosphatidyl serine, and can play roles in the blood hemostasis, thrombosis, cancer and inflammation similar to platelets [12, 13]. PMPs are involved in thrombotic problems and cardiovascular complications in T2DM. Coagulation activities of PMPs are 50 to 100 times more than active platelets; hence, they are considered as potential targets for the reduction of atherosclerosis development in diabetes [14].

Desensitization might be generally considered as an adaptive mechanism that is applied by some kinds of cells to regulate their responses when they are exposed to continuous stimulations. Desensitization of platelets can be diagnosed according to the decline of activation response of cells and can be regulated by intracellular signaling mechanisms [15]. After continuous mild stimulation with endogenous ADP, platelets indicate a declined sensitivity to be activated by strong ADP stimulations [16]. This phenomenon can be also observed following long term treatments with drugs that act as agonist receptors [17]. Desensitization of platelets occurs in vivo after major surgeries and may be involved in post-surgery bleeding events. Desensitization can be also seen in vitro, in particular for suspended platelets in buffers as a replacement for plasma. This state is temporary and disappears after enzymatic degradation of the available ADP in the environment [18]. The ATP/CD39 diphosphohydrolase on the surface of healthy vessels prevents the activation of platelets under normal conditions. The function of this enzyme is impaired in clinical conditions that are associated with vascular damages including diabetes [19]. G-protein dependent ADP receptors, P2Y1 and P2Y12 are involved in the desensitization of platelets after the long-term interaction with ADP. Desensitization process is complex and includes the receptor phosphorylation, separation of G-proteins and internalization of receptors [20]. Considering pro-thrombotic conditions in patients with T2DM, the desensitization of platelets to the ADP might be an adaptive response preventing major thrombotic events. In the present study, platelet samples of patients with T2DM were evaluated for determining the activation responses as well as amount of PMPs formation after mild and strong ADP stimulations. Accordingly, we sought to determine whether an increase in thrombotic events in the patients with type 2 diabetes was due to an elevation in the sensitivity of platelets to ADP or because of increased formation of platelet micro particles.

## Methods

### Participants

In the present study, 40 patients with T2DM referred to the central medical laboratory of Kashan, and 35 healthy volunteers were included after receiving their consent. All participants were non-smokers, and did not consume any anti-platelet drugs at least for two weeks. The inclusion criteria of T2DM were as follows: FBS ≥ 126 mg/dl; OGTT≥200 mg/dl; hemoglobin A1C ≥ 6.5%; and patients under the medication and insulin therapy. The patients were asked for fasting and non-use of antiplatelet drugs before sampling. Renal function was normal in all patients and they still had no kidney symptoms. Sampling of participants (both patients with T2DM and healthy controls) was randomly performed. After participants’ qualification for inclusion criteria, they completed informed consent forms. Table 1 presents general characteristics of T2DM and controls. Ethical approval of study was obtained from the ethics committee of Shahid Beheshti University of Medical Sciences.

**Table 1:** General characteristics of T2DM and controls

	T2DM (*n* = 40)	Control (*n* = 35)
Age (years)	55.41 ± 7.23	54.61 ± 8.62
Diabetic duration (years)	6.08 ± 2.64	None
Male	22	17
Female	18	18
Fasting Blood sugar (mg/dl, mean)	145 ± 8.0	92 ± 5.0
HbA1c (%, mean)	8.36 ± 2.35	5.47 ± 0.22
OGTT (mg/dl, mean)	275 ± 14.35	170 ± 12.35
Microvascular complications	None	None
Macro vascular complications	None	None
Treatment		None
Insulin, *n* (%)	15	None
Oral anti-diabetic agents, *n* (%)	19	None
Insulin+Oral anti-diabetic agents, *n* (%)	6	None

### Blood samples collection and the PRP preparation

The collected blood samples into tubes containing sodium citrate 3.8% as the anticoagulant were subjected to the soft centrifugation to obtain the platelet rich plasma (PRP).

### Experimental treatments

Platelets in PRP samples were subjected to the mild stimulation (low dose) with ADP (1 μM) for an hour, or to the strong stimulation (high dose) with ADP (10 μM) for 10 min or both in a sequential order (Low+ High) [15, 21]. A sample remained non-stimulated (Resting platelets or baseline stage). Paraformaldehyde (PFA) was added to all platelet samples at a final concentration of 1% to stop the stimulation. The process could lead to the desensitization of platelets to ADP which was then verified by the flow cytometric analysis. Four flow cytometry tubes were considered for each PRP sample in order to study the impact of each concentration of ADP on platelets.

### Flow cytometry analysis

**Fig. 1:**
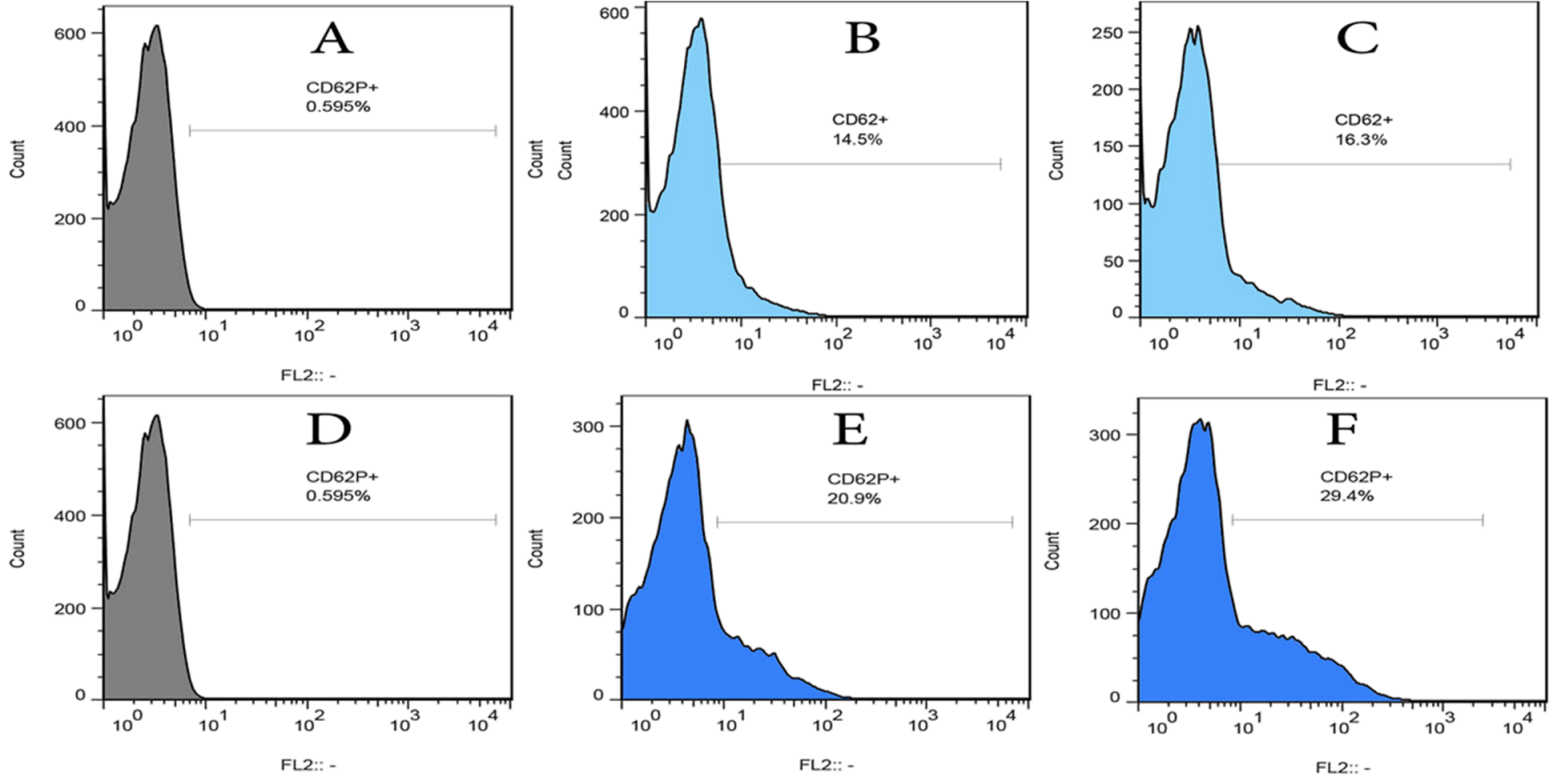
Expression of CD62P in histogram charts in FL2 channel (Antibody for CD62P) after gating platelets in a FSC for SCC chart, then analyzing in a histogram chart in FL2 channel. **a**, **d**: Isotype controls were used for each flow cytometry run. **b**,**c**: **b** is a histogram of low-dose ADP treatment in control; and **c** is a histogram of Low + High dose ADP treatment in control. **e**, **f**: **e** is a histogram of Low dose ADP treatment in Diabetes and **f** is a histogram of Low + High dose ADP treatment in Diabetes

**Fig. 2:**
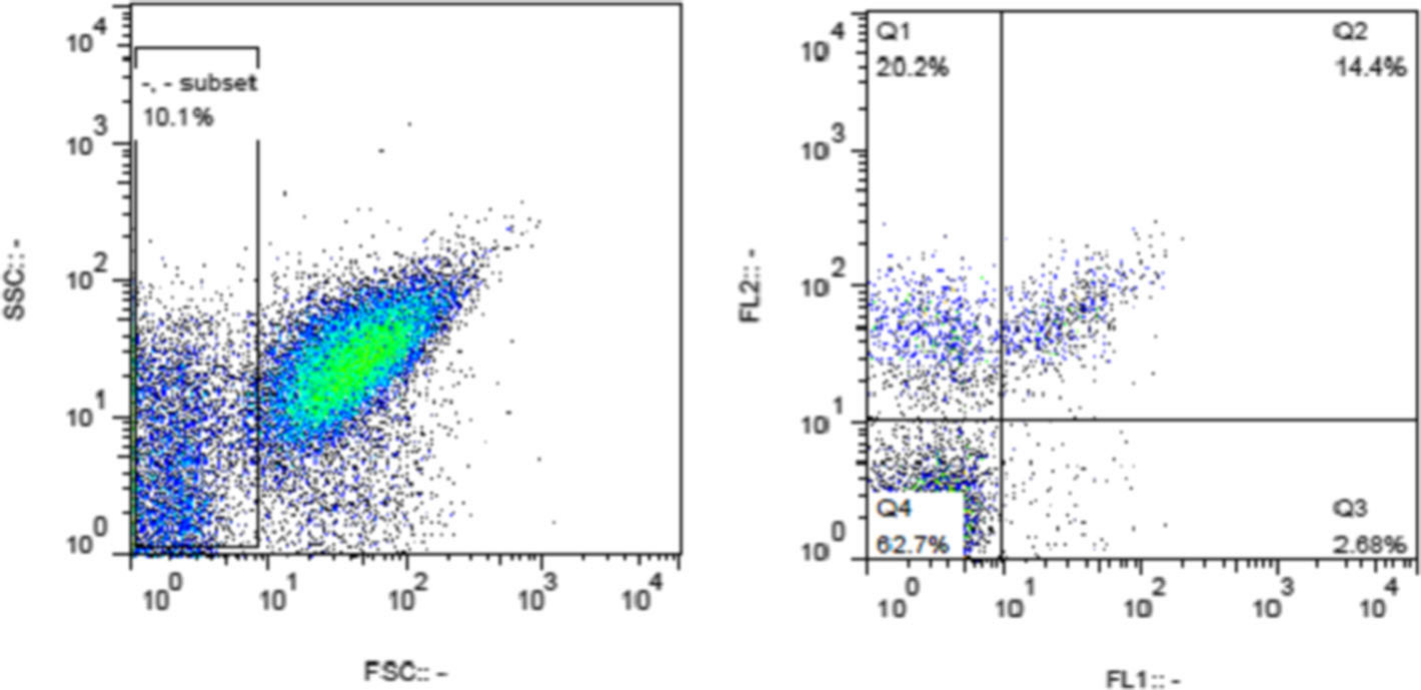
Platelets mico paticles gating. The micro particles were gated as events less than one micron and then the micro particles were specified considering the FL1 channel (Antibody against Annexin-v) and FL2 channel (Antibody against CD41)

The flow cytometry analysis of samples was conducted using CyFlow®Space, Partec GmbH (Germany). Four tubes were considered for each PRP sample. The first tube contained resting platelets (Baseline) without adding any ADP. The second tube contained treated platelets at a low concentration of ADP (Low) incubated for an hour; and the third tube contained treated platelets in a low dosage of ADP and incubated for an hour, and then treated with a high dosage of ADP and incubated for 10 min (Low + High). The forth tube contained platelets which were only treated at a high concentration of ADP (High) for 10 min. The flow cytometry measurement was conducted for each stage in two separate tubes. One for incubation with the CD62P specific antibody conjugated with RPI (ABCAM, UK) and the other containing CD41 specific antibody conjugated with RPI (DAKO, Germany) and an Annexin-v specific antibody conjugated with FITC (TREVIGEN, US). Incubation of samples with antibodies was implemented an hour at the room temperature with mild shaking. The expression of CD62P and counting CD41 + -Annexin-v + micro particles were measured in each tube. The expression of CD62P was done after gating the platelets in a FSC for SCC chart, and then analyzed in a histogram chart in FL2 channel (Antibody for CD62P). Isotype controls were used for each flow cytometry run (Fig. 1). The micro particles were gated less than a micron in order to gate the platelet micro particles at the first stage of the FSC and SCC chart, and then the micro particles were specified considering the FL1 channel (Antibody against Annexin-v) and FL2 channel (Antibody against CD41)(Fig. 2).

The extent of expression for CD62P was reported in percentage as the mean fluorescent intensity (MFI); and the number of measured platelet micro particles was stated as their number in the microliter. The derived data from the flow cytometry was then analyzed using Flowjo software (FlowJo 7.6.2, Engine 2.97000. OS version: Windows 7).

### Statistical analysis

The MFI was calculated for a large sample of platelets (20,000 platelets per samples) for each flow cytometric sample. SPSS 16.0 was used with a *P* value < 0.001 in the expression of CD62P; and *P*-value < 0.001 in micro particles was considered significant. According to the Kolmogorov-Smirnov test, the population distribution was normal at all stages of treatment with ADP in both healthy people and the T2DM group (*p* > 0.05).

## Results

**Fig. 3:**
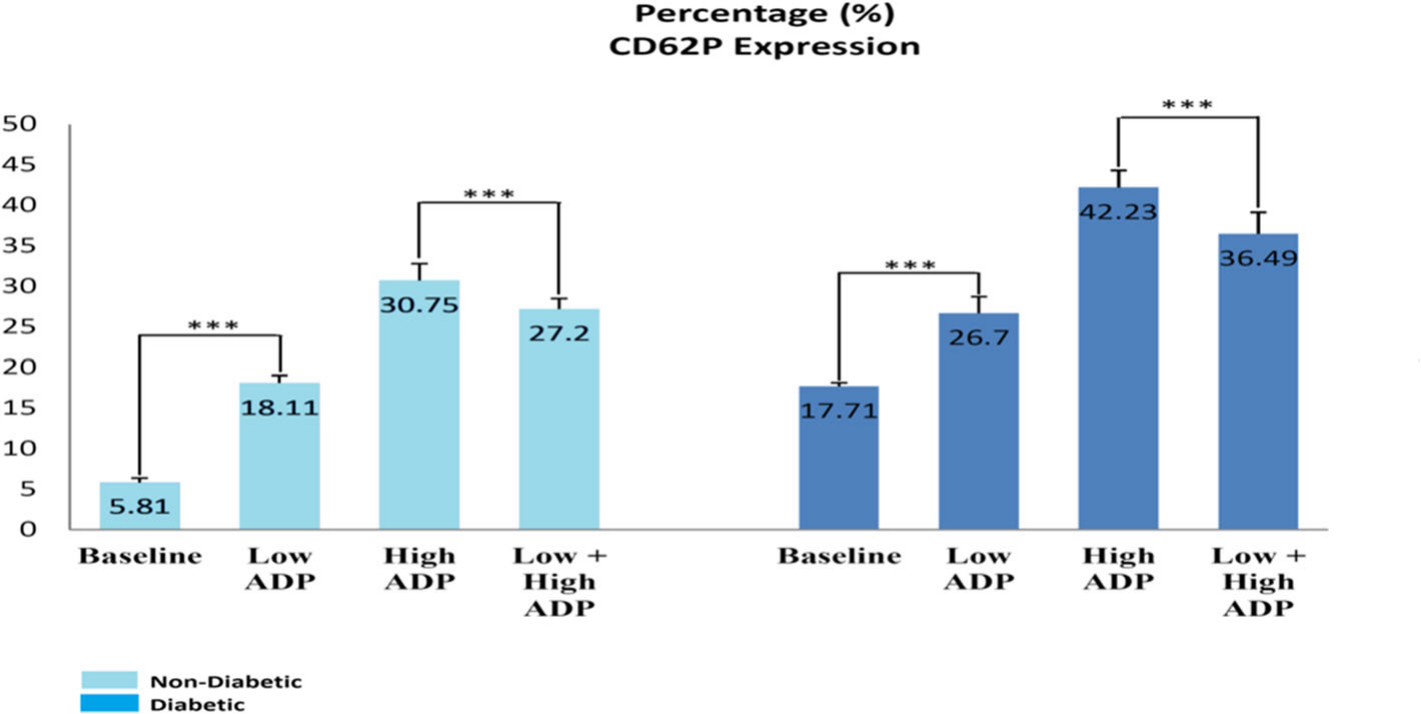
Expression of CD62P at experimental stages in Diabetic and non- Diabetic samples. The extent of expression for CD62P was reported in percent as mean fluorescent intensity (MFI) with a P = value < 0.003. ^***^ *P* < 0.001 represents significant changes from Non- Diabetic control

**Fig. 4:**
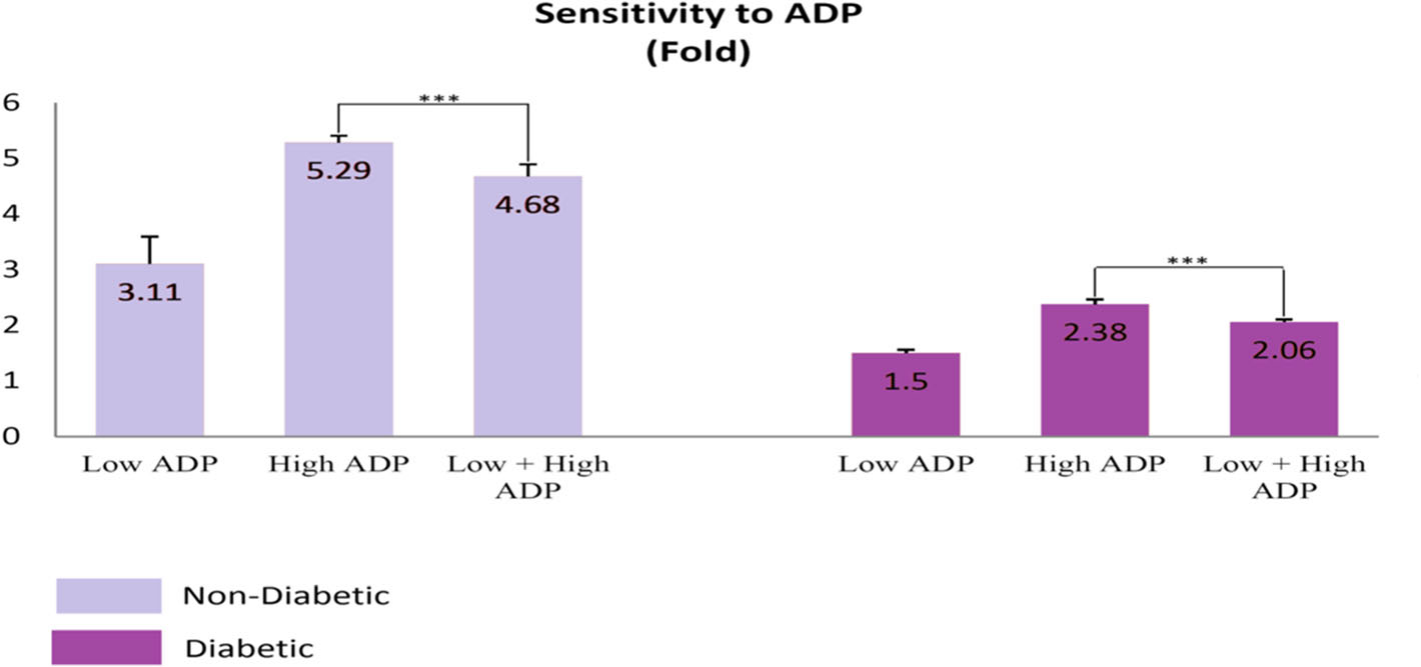
The extent of platelets sensitivity and their irritability to ADP. The more the platelets are in resting, the more irritability it has to ADP. The mild agonist stimulation of the platelets (Low + High ADP) led to reduced irritability of the platelets to ADP. *** P < 0.001 represents significant changes from Non- Diabetic control

The flow cytometry analysis indicated that platelets of patients with T2DM had a higher expression of CD62P at the resting stage (baseline), and they expressed a higher level of CD62P even in the absence of exogenous ADP. In the second to forth tube of PRP, this increased expression of CD62P was greater in patients with T2DM in comparison with healthy participants; and the difference was due to the pre-activity of platelets at the baseline stage (Fig. 3). According to the comparison of high-dose tubes and High+ Low dose tubes, the mild agonist treatment led to the lower CD62P induction and reduced reactivity of platelets. The lower expression of CD62P might be due to the desensitization phenomenon as a result of mild agonist stimulation (Fig. 3). The phenomenon played a significant role in turning off signal transduction pathways in platelets. This lower reactivity of platelets as a result of mild agonist stimulation was seen in both groups; however, the reduction of CD62P expression was higher in patients with T2DM. Furthermore, the extent of platelets sensitivity and their irritability to ADP was calculated and reported as the Fold. The term “Fold” was considered to better show levels of sensitivity and irritability of platelets relating to ADP. To this end, the CD62P expression level at each stage of the agonist treatment was divided by the CD62P expression level at the baseline (the stage before the start of agonist treatment) and the resulting number was reported as Fold and it was accordingly charted (Fig. 4). Sensitivity and irritability of platelets to ADP were lower in diabetic samples, but higher in normal samples. In other words, the more platelets were in rest, the more irritability it had to ADP. On the other hand, the mild agonist platelet stimulation, which might cause the desensitization of platelets, led to the reduced irritability of platelets to ADP.

**Fig. 5:**
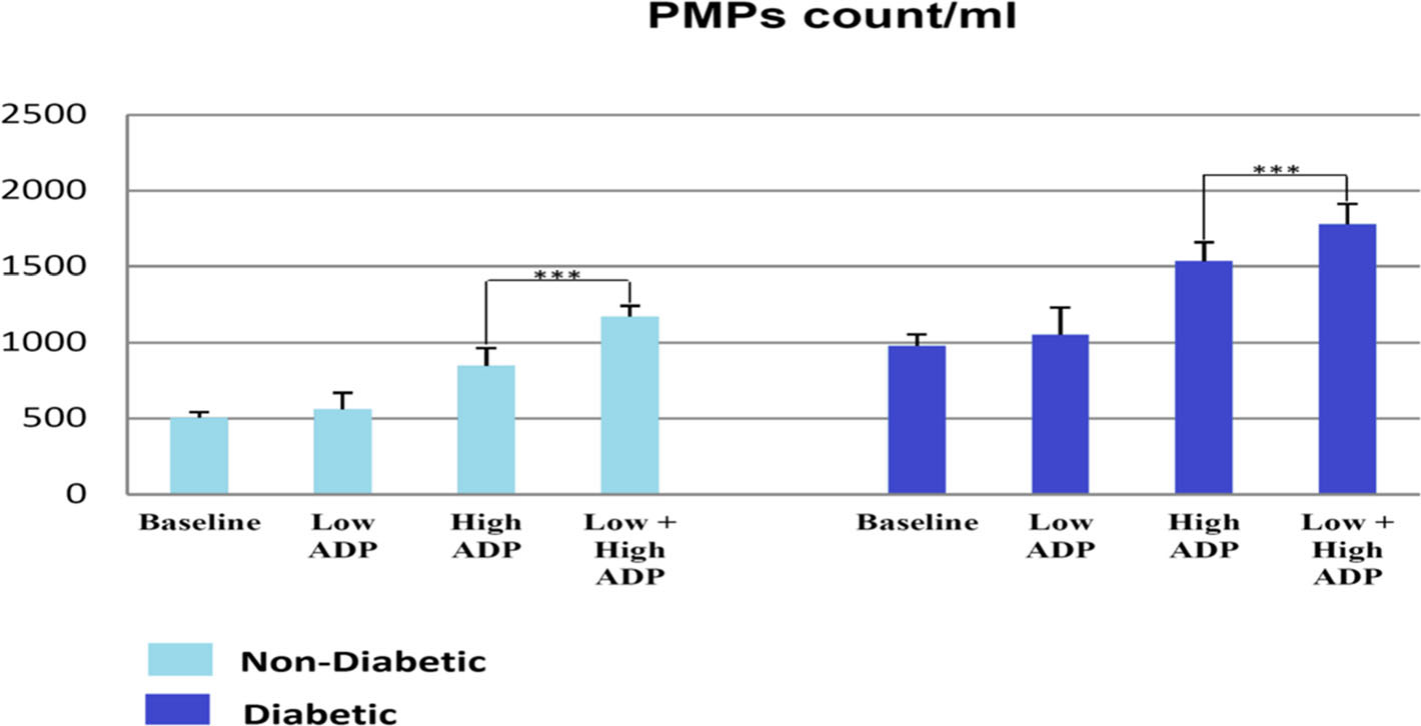
The number of CD41 + -Annexin-v + platelet micro particles for each tube. The counted number of platelet micro particles in all four tubes was higher for the diabetic platelets. The platelets, which were treated with a low dosage of ADP and afterwards were treated with High dosage of ADP, formed more micro particles comparing to ones that were treated with only high dosage of ADP. Despite the fact that platelets, which first had mild agonist treatment and then were treated with high dosage of ADP actually had lower CD62P induction, they were likely to form more platelet micro particles. *** *P* < 0.001 represents significant changes from Non- Diabetic control

The number of CD41 + -Annexin-v + platelet micro particles was separately counted for each tube after the observation of extent of expression of CD62P for each tube (Fig. 5). The counted number of platelet micro-particles in all four tubes was higher for diabetic platelets; and the formation of micro platelets was higher in these patients than healthy individuals. Micro-particles were also formed in baseline platelet samples due to the activation of platelets during the sampling process and preparation stages. However, there were considerable findings about micro-particles indicating that the highest rate of formation of platelet micro-particles belonged to a tube with High+ Low dose ADP rather than a tube with high ADP.

On the contrary to the expectation, the platelets, which were treated with low dosage of ADP and then with high dosage of ADP, created more micro particles than ones that were treated with only high dosage of ADP. Therefore, despite the fact that platelets, which first had mild agonist treatment and then were treated with high dosage of ADP, had lower CD62P induction, they were likely to create more platelet micro-particles.

## Discussion

Given the obtained data from the flow cytometry, it can be concluded that platelets express higher CD62P levels in patients with type 2 diabetes mellitus, and are pre-active at the baseline stage without the hexogen agonist treatment since the beginning as this state of platelets is related to the existing metabolic environment in patient with type 2 diabetes mellitus. Platelets are quite sensitive and responsive to environmental changes. The rate of glucose concentration in platelets indicates the rate of extracellular glucose concentration because glucose is not dependent on insulin in order to enter platelet [22–24]. Chronic and long-term hyperglycemia is particularly recognized as a major factor for the activation of platelets and formation of pre-active platelets in patients with type 2 diabetes mellitus [25–27]. In the present study, the treatment of PRPs with mild agonist ADP stimulation led to the lower induction of CD62P expression and reduced reactivity in both diabetic and control platelets probably due to the desensitization phenomenon. The extent of CD62P expression was lower in a tube, which first had a mild agonist stimulation, and it was then treated with a high dosage of ADP than a tube which was solely treated with a high dosage of ADP, and thus it can be concluded that platelets decreased the response to agonist under the effect of desensitization phenomenon that could help platelets that were constantly exposed to agonist in a diabetes metabolic environment [28]. This reduced response is higher among diabetic platelets and leads to the higher reduction of expression for CD62P. In the diabetes metabolic environment, platelets are constantly exposed to the produced ADP by platelets that are activated in such an environment and under hyperglycemia. Based on findings of the present research, this phenomenon is effective for reducing the activation of platelets in response to ADP. Platelets are pre-active in the patients with type 2 diabetes mellitus and display the lower irritability to ADP either in low or high dosages. This reduction of sensitivity and irritability of diabetic platelets might be due to the desensitization of diabetic platelets in diabetic vessels and in constant stimulation by ADP, thereby leading to the reduced response of platelets to ADP. However, the sensitivity and irritability of platelets of non-diabetic individuals to ADP are always higher and express more rates of CD62P throughout the agonist treatment. The mild agonist stimulation of platelets (for both groups) led to reduced irritability of platelets to ADP. According to platelet micro-particles, findings indicated that if platelets, which were already exposed to the mild agonist stimulation and desensitized under diabetic vascular conditions, were exposed to high dosage of ADP, for instance, to a thrombotic clot, they would produce more amounts of micro-particles. High rate and systematic production of platelet micro-particles was considered as a pro-inflammatory mediator and pathological factor [29]. Chronic hyperglycemia and pre-active platelets with high expression rates of CD62P among patients with type 2 diabetes mellitus predisposed the patients to atherosclerosis as an important complication among such patients. There is the evidence for roles of platelets in the atherosclerosis, which is intermediated by production of platelet micro-particles, that can be considered as a prognostic marker for atherosclerotic cardiovascular diseases such as diabetes [30, 31]. Study limitations included small sample sizes and time limit. It is recommended that this study should be done with a larger sample size in several clinics. Furthermore, responses of platelets to natural anti-aggregation material such as PGI2 and NO are reduced among these patients; and all items of pathogenesis of atherosclerosis are also involved in this disease [32]. Based on the derived data from this research, the response of platelets and their irritability to ADP indicated a reduction among patients; and such reduced sensitivity to ADP along with reduced response to *PGI2* and *NO* might be according to the occurrence of atherosclerosis in the patients. Considering structural characteristics and sizes of platelet micro-particles, their local production made them strong tools in platelet-cellular communications for transfer of bioactive molecules with platelet origin such as growth factors and other signaling molecules and miRNAs [33]. It occurs if they act as pro-inflammatory mediators and pathological factors in different pathological situations when they are systematically and abundantly produced, thereby leading to the progress of atherosclerosis and thrombotic complications [34, 35]. According to the research data, the platelets, which had the lower reactivity, produced more micro-particles. Such micro-particles in a systematic production brought thrombotic complications; and the phenomenon might be involved in diabetic complications as well. Diabetic ulcers are the most common and serious complications of diabetes. Ulcer healing is a complex biological and dynamic process consisting of four stages, namely hemostasis, inflammation, proliferation and remodeling. This process includes various types of cells, extracellular compounds, growth factors and cytokines all of which are affected by diabetes type 2 diseases and lead to delayed healing of ulcers [36, 37]. Since the mild platelet stimulation, which causes developing desensitization, leads to the reduced reactivity of platelets, it may be a factor for facilitating such a late healing of diabetic ulcers.

## Conclusion

Under mild agonist stimulation conditions and despite the high expression of CD62P, platelets had a low sensitivity to ADP probably due to the desensitization that helped platelets to adapt to their metabolic environment. Therefore, such lower reactivity of platelets to ADP along with reduced sensitivity to PG- I2 and NO could play roles and be involved in increasing the occurrence of atherosclerosis among patients with type 2 diabetes mellitus. On the other hand, platelets were likely to produce more micro-platelets under the influence of a mild agonist stimulation that could be involved in thrombotic complications of diabetes.

## Data Availability

All data generated or analyzed during this study are included in this published article and are presented within the additional supporting files as the name “Availability of data”.

## Abbreviations

ADP: Adenosine diphosphate
FBS: Fasting Blood sugar
HbA1c: Glycosylated hemoglobin A1c
NO: Nitric Oxide
OGTT: Oral glucose tolerance test
PGI2: Prostaglandin I2
PMPs: Platelet micro particles
